# Transcriptome and Physiological Analysis of Rootstock Types and Silicon Affecting Cold Tolerance of Cucumber Seedlings

**DOI:** 10.3390/plants11030445

**Published:** 2022-02-06

**Authors:** Heng Luan, Chenxu Niu, Xinmiao Nie, Yan Li, Min Wei

**Affiliations:** 1 College of Horticultural Science and Engineering, Shandong Agricultural University, Taian 271018, China; 2019110265@sdau.edu.cn (H.L.); 2021010073@sdau.edu.cn (C.N.); 2019110260@sdau.edu.cn (X.N.); 2020010076@sdau.edu.cn (Y.L.); 2 Scientific Observing and Experimental Station of Environment Controlled Agricultural Engineering in Huang-Huai-Hai Region, Ministry of Agriculture and Rural Affairs, Taian 271018, China; 3 State Key Laboratory of Crop Biology, Taian 271018, China; 4 Collaborative Innovation Center of Fruit & Vegetable Quality and Efficient Production in Shandong, Taian 271018, China

**Keywords:** cucumber, cold tolerance, rootstock, silicon, transcriptome

## Abstract

Cucumbers grafted on rootstocks with different de-blooming capacity show varying levels of cold tolerance. The content of fruit bloom correlates with its silicon-metabolizing capacity, and rootstock grafting can alter not only the cold tolerance but also the silicon-metabolizing capacity of the scion. The molecular mechanisms responsible for resistance due to rootstocks and silicon and the pathway that affects cold tolerance, however, remain poorly understood. Therefore, we performed physiological and transcriptome analysis to clarify how rootstock types and silicon affect cold tolerance in cucumber seedlings. Then, we randomly selected eight differentially expressed genes (DEGs) for quantitative real time PCR (qRT-PCR) analysis to proof the reliability of the transcriptome data. The results showed that silicon can enhance the cold tolerance of cucumbers by boosting the phenylpropanoid metabolism, and rootstock grafting can boost the active oxygen scavenging ability and synthesis level of hormones in cucumbers and maintain the stability of the membrane structure to enhance cold tolerance. The difference in cold tolerance between the two rootstocks is because the cold-tolerant one has stronger metabolic and sharp signal transduction ability and can maintain the stability of photosynthesis, thereby contributing to the stability of the cellular system and enhancing tolerance to cold.

## 1. Introduction

Cucumber (*Cucumis sativus* L.) is a major vegetable crop cultivated in solar greenhouses in China and is sensitive to cold [1]. Grafting is an essential agronomic technique to enhance the cold tolerance of cucumbers [2], especially for cucumber production in greenhouses in northern China. Previously, black-seeded pumpkins were mainly used as a rootstock for cucumber grafting, as they exhibit strong resistance, high yield, and other advantages [3]. However, the commercial value of black-seeded-pumpkin-grafted cucumber has decreased due to fruit bloom [4]. In recent years, cucumbers with less fruit bloom and brighter surface have become more popular in the market [5]. To reduce the fruit bloom and increase the commercial value, the proportion of cucumber varieties grafted on white-seed rootstock with strong de-blooming ability has been increasing in production.

Studies have shown that black-seeded pumpkin rootstock can not only enhance the resistance of cucumbers to cold [6] but also the resistance to salt [7,8] and heavy metal ion stress [9]. The ability of grafted cucumbers to tolerate cold also differs significantly between pumpkin rootstocks with different de-blooming capacity. Our previous study showed that cucumbers grafted on rootstocks with a strong de-blooming ability were relatively less tolerant to the cold [10]. Although black-seeded pumpkin rootstocks were not conducive to the removal of bloom of grafted cucumbers, they were more tolerant to cold than white-seeded-grafted rootstocks. This led to a keen interest in determining whether there is a correlation between the de-blooming ability and the cold tolerance of grafted cucumbers.

The amount of fruit bloom depends on the distribution characteristics of silicon absorption, and the content of fruit bloom of cucumber increases or decreases significantly with the increase or decrease in silicon [11,12]. Our experiments have also proven the effect of rootstocks with different de-blooming capacity on the bloom formation of grafted cucumber and the relationship between bloom formation and silicon absorption and metabolization [4]. Because silicon is a beneficial element for crop growth and enhances the resistance to adversity-induced stress [13], we speculate that the ability of cucumbers to tolerate cold may be related to the metabolic properties of silicon absorption and metabolization.

Lipid metabolism and a range of redox processes are reported to play a significant role in the response of crops to cold stress [14]. Lipid metabolism, including membrane lipid and fatty acid metabolism, highly influences the response to cold stress [15,16]. Phenylpropanoid metabolism [17], phytohormone signaling [18], and transcription factors [19] have also been reported to regulate cold tolerance in crops. Transcriptome analysis has also been used to understand cold tolerance in horticultural crops such as tomato [20], watermelon [21], and eggplant [22]. However, to date, studies at the transcriptional level on how rootstock types and silicon affect cold tolerance in cucumber and the underlying causes of differences in cold tolerance have not been reported.

In this work, we used ‘Xintaimici’ self-rooted cucumbers, the strong de-blooming rootstock ‘Huang Chenggen No. 2 (*C. moschata* Duch × *C. moschata* Duch)′-grafted cucumbers, and the weak de-blooming rootstock ‘Yunnan figleaf gourd (*Cucurbita ficifolia* Bouche)’-grafted cucumbers as examined materials, and silicon was added to the root system. Hence, in addition to physiological experiments, the transcriptomics method was used to reveal the mechanisms by rootstock types and how silicon affects cold tolerance in cucumber. By comparing the differences in gene expression between the seedlings under cold conditions, we provide a molecular framework for understanding the cold tolerance mechanism of cucumber and lay a foundation for further research.

## 2. Results

### 2.1. Physiological Response of Cucumber Seedlings under Cold Stress

**Figure 1:**
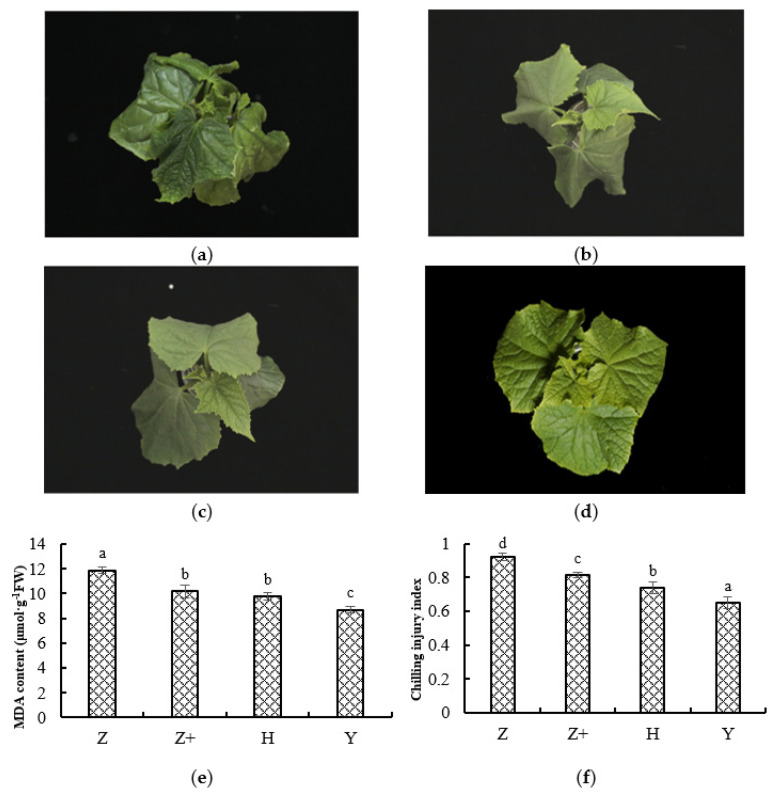
Determination of physiological characteristics under cold treatment. The phenotypes of cucumber seedlings were self-rooted cucumber (**a**, Z), self-rooted cucumber with silicon addition (**b**, Z+), ‘Huangchenggen No. 2′-grafted cucumber (**c**, H) and ‘Yunnan figleaf gourd’-grafted cucumber (**d**, Y). MDA content (**e**) and chilling injury index (**f**) of cucumber seedings under cold treatment for 7 days, respectively. Three biological replicates were used, with six plants per replicate. Mean value ± standard deviation were represented, and different letters (a, b, c) indicate that they were significant differences at a level of 0.05, according to Duncan’s test.

To determine the effect of grafting and addition of silicon on the cold tolerance of cucumbers, we observed the phenotype and measured the changes of malondialdehyde (MDA) content and chilling injury index of cucumber seedlings under cold stress. After cold stress for 7 days, cucumber seedlings began to show leaf curling and wilting, as well as growth inhibition (Figure 1a–d). In addition, MDA content was measured to investigate physiological variation in cucumber seedlings, at same time, the chilling injury index of cucumber seedlings was evaluated after 7 days of cold treatment. The MDA and the chilling injury index contents of grafting and silicon-addition seedlings were significantly decreased compared with self-rooted seedlings after 7 days of cold treatment (Figure 1e,f). These results indicate that grafting and silicon addition apparently enhance the cold tolerance of cucumbers.

### 2.2. Transcriptome Sequencing Data and Quality

To visualize the quality of library construction and sequencing of the samples, we performed a quality analysis of the data and detected a total of 47,441,526 to 49,053,480 reads (Table S1). The GC content of all 12 leaf samples exceeded 43%, the average base mass of Q20 was greater than 97%, and the average base mass of Q30 was greater than 92%, which indicated that the transcriptome sequencing quality was good. A comparison was performed with the cucumber genome database as the reference genome, and the comparison rate of each sample with the reference genome reached more than 95%, indicating that the selected reference genome has normal comparison results and met the requirements of gene function annotation and analysis.

### 2.3. Statistics of DEGs

**Figure 2:**
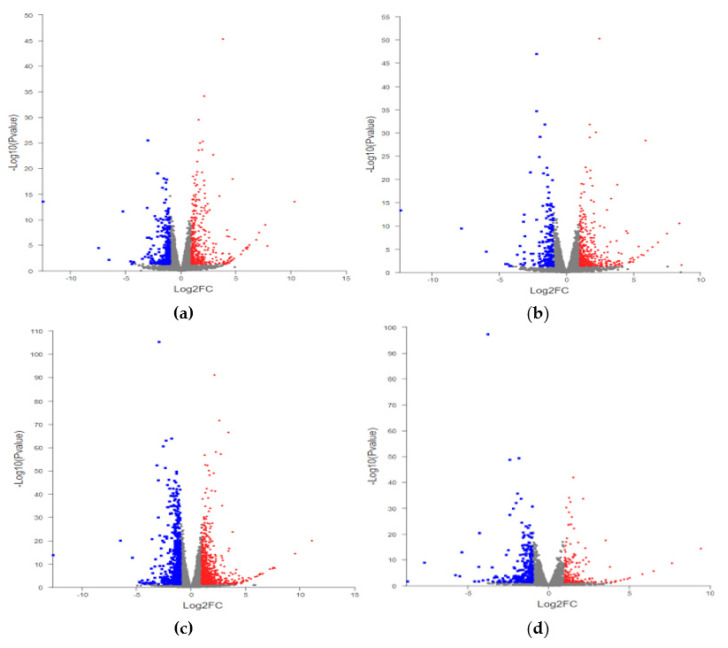
Volcano Polt of differentially expressed genes (DEGs). (**a**) Comparsion of self-root cucumber with silicon addition (Z+) and self-rooted cucumber (Z); (**b**) comparsion of ‘Huangchenggen No. 2′-grafted cucumber (H) and self-rooted cucumber (Z); (**c**) comparsion of ‘Yunnan figleaf gourd’-grafted cucumber (Y) and self-rooted cucumber (Z); (**d**) comparsion of ‘Yunnan figleaf gourd’-grafted cucumber (Y) and ‘Huangchenggen No. 2′-grafted cucumber (H). The red column indicates the number of upregulated genes, whereas the blue one means downregulated genes.

Under cold treatment, silicon induced differential expression of 602 genes as compared with the control (Figure 2a, Table 1). Grafted cucumbers from the ‘Huang Chenggen No. 2′ rootstock had 596 DEGs (Figure 2b, Table 1), whereas grafted cucumbers from the ‘Yunnan figleaf gourd’ rootstock had 1349 DEGs (Figure 2c, Table 1). There were 461 DEGs between the cucumbers grafted from the two types of rootstock (Figure 2d, Table 1).

### 2.4. Functional Annotation of DEGs

**Figure 3:**
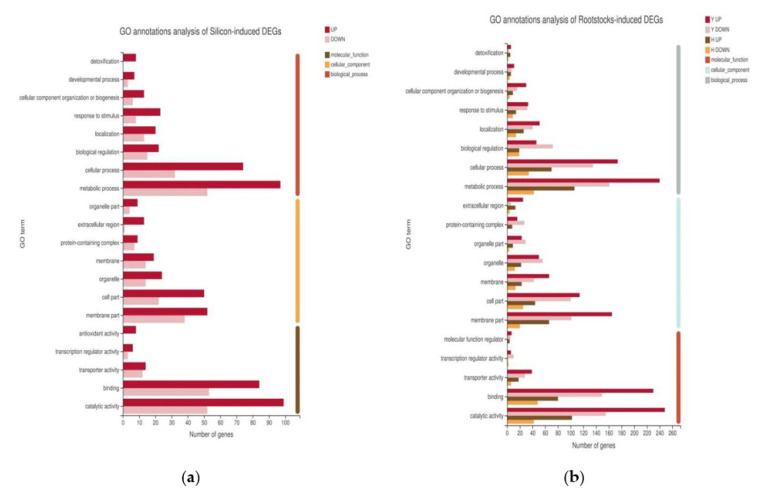
GO annotation analysis of DEGs for (**a**) the comparison of self-rooted cucumber with silicon addition (Z+) and self-rooted cucumber (Z), and (**b**) the comparison of two types of rootstock-grafted Cucumber (H and Y) with self-rooted cucumber (Z).

To further investigate the biological functions of rootstock grafting and silicon-induced DEGs under cold stress, we performed a functional annotation of DEGs via Gene Ontology (GO). By comparing with the GO Consortium, we found that the silicon-induced DEGs were similar to the rootstock-grafting-induced DEGs in terms of functional classification, mostly in the metabolic process, catalytic activity, membrane part, binding, and cellular process categories (Figure 3). We also found no difference between the upregulated and downregulated DEGs. These results indicate that most DEGs in cucumbers are involved in metabolic processes, cellular activity, membrane structure, and catalytic activity, suggesting that cold treatment mainly affects physiological metabolism, membrane structure, and cellular activity in cucumbers.

### 2.5. KEGG Enrichment Analysis of Silicon-Induced DEGs

**Figure 4:**
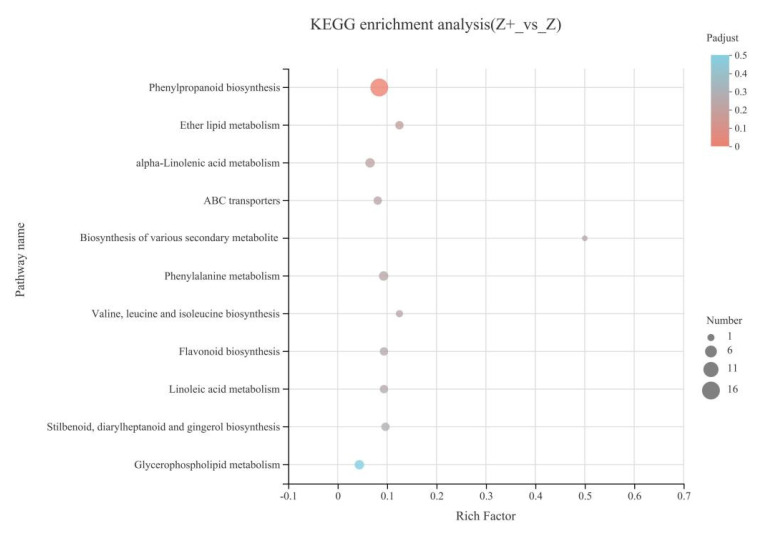
KEGG enrichment analysis of DEGs for the comparison of self-rooted cucumber with silicon addition (Z+) and self-rooted cucumber (Z), and “Benjamini and Hochberg” test was used to detect significantly enriched KEGG terms (FDR < 0.05, *p* < 0.05).

To clarify the metabolic pathways involved in silicon-induced DEGs and their functions, we performed the KEGG enrichment analysis of DEGs in the self-rooted cucumbers. The results showed that the phenylpropanoid biosynthesis pathway was significantly enriched (Figure 4). It is hypothesized that the phenylpropanoid metabolic pathway plays a significant role in cold tolerance of cucumbers, and is induced by silicon.

### 2.6. KEGG Enrichment Analysis of Rootstock-Grafted-Induced DEGs

**Figure 5:**
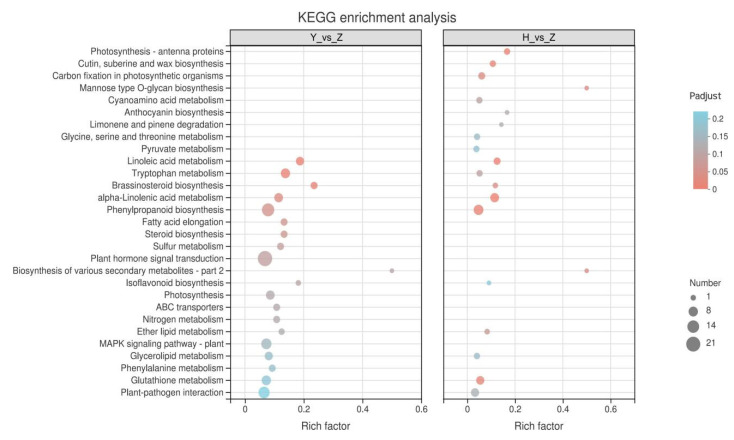
KEGG enrichment analysis of DEGs for the comparison of two kinds of pumpkin-grafted cucumber (H and Y) with self-rooted cucumber (Z), and the “Benjamini and Hochberg” test was used to detect significantly enriched KEGG terms (FDR < 0.05, *p* < 0.05).

To clarify the metabolic pathways involved in rootstock-grafting-induced DEGs and their functions, we performed the KEGG enrichment analysis of DEGs in ‘Huang Chenggen No. 2′ and ‘Yunnan figleaf gourd’-grafted cucumbers. The comparison revealed that alpha-Linolenic acid metabolism and Linoleic acid metabolism associated with membrane lipid metabolism, the Brassinosteroid biosynthesis pathway associated with hormones, and the Glutathione metabolism pathway associated with reactive oxygen species (ROS) metabolism were enriched in grafted cucumbers from both rootstocks (Figure 5). 

To investigate the mechanisms responsible for differences in cold tolerance, we compared the metabolic pathways of DEGs induced by two types of rootstocks. DEGs induced by ‘Yunnan figleaf gourd’-grafted cucumbers were found to be enriched in phenylalanine metabolism, MAPK signaling pathway, and primary metabolic processes such as Nitrogen metabolism and Sulfur metabolism as compared with those induced by ‘Huang Chenggen No. 2′ rootstock (Figure 5). 

### 2.7. Screening for Transcription Factors

To elucidate the expression of transcription factors under cold stress, we conducted a screening of transcription factors by analyzing the DNA-binding domain information of the genes. By performing functional annotation of genes, we screened 26 transcription factors induced by silicon (Table S2), 29 transcription factors induced by grafting on ‘Huang Chenggen No. 2′ rootstock (Table S3), and 49 transcription factors induced by grafting on ‘Yunnan figleaf gourd’ rootstock (Table S4). Among them, ETHYLENE-RESPONSIVE TRANSCRIPTION FACTOR 54 (*ERF54*) was upregulated in both silicon addition and rootstock-grafting cucumbers, and it was speculated that this transcription factor might be involved in regulating the cold tolerance of cucumber.

MYB DOMAIN PROTEIN (MYB) transcription factors *MYB44* were specifically upregulated after silicon addition, suggesting that they may be involved in the regulation of cold tolerance in cucumber and are influenced by silicon. The differential expression of *WRKY48* and other WRKY transcription factors in grafted cucumbers from both rootstocks suggests that the WRKY transcription factor family is involved in the regulation of cold tolerance in grafted cucumber. Among these transcription factors, *WRKY70* was specifically expressed in the cold-tolerant ‘Yunnan figleaf gourd’-grafted cucumber, which may be associated with the different tolerance among two types of rootstocks. Other transcription factors such as BASIC HELIX-LOOP-HELIX (bHLH) family members bHLH62 and bHLH137 or the NAC DOMAIN CONTAINING PROTEIN NO APICAL MERISTEM (NAM), ATAF1, ATAF2 and CUP-SHAPED COTYLEDON 2 (CUC2) were also differentially expressed. 

### 2.8. Validation of the Transcriptome Data

**Figure 6:**
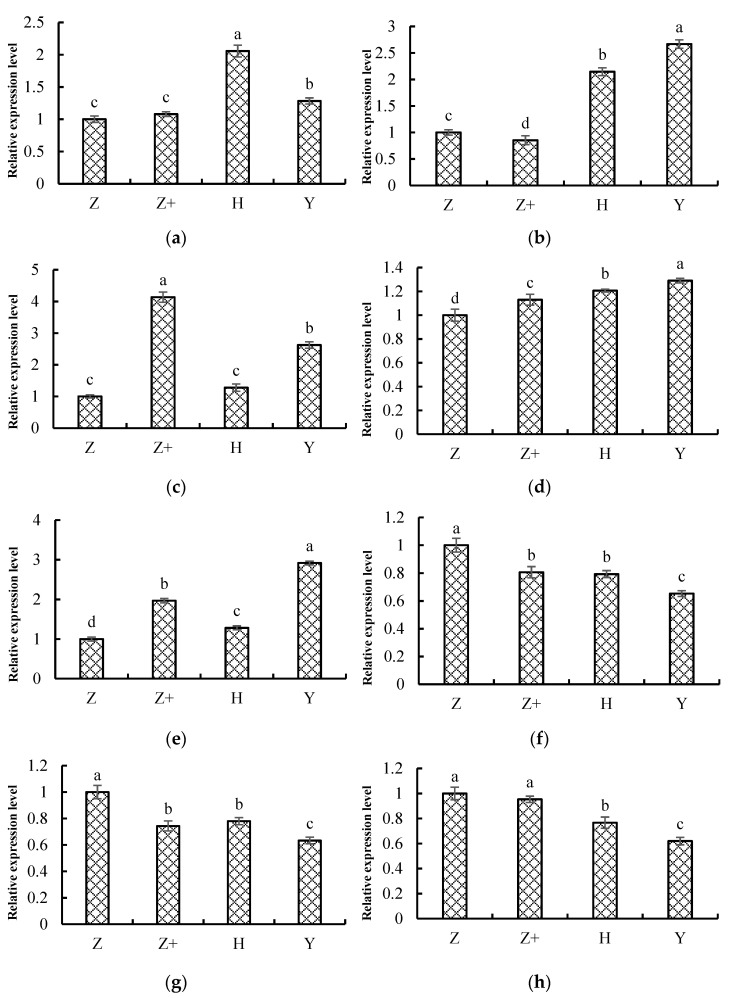
Transcriptome sequencing confirmation by qRT-PCR. The eight genes were randomly selected. Different treatments represent: self-rooted cucumber (Z), self-rooted cucumber with silicon addition (Z+), ‘Huangchenggen No. 2′-grafted cucumber (H) and ‘Yunnan figleaf gourd’-grafted cucumber (Y). Genes were (**a**) ethylene responsive transcription factor ERF054-like; (**b**) WRKY transcription factor 48; (**c**) phenylalanine ammonia-lyase; (**d**) peroxidase 31; (**e**) ABC transporter B family member 2; (**f**) glutathione S-transferase U8; (**g**) chlorophyll a-b binding protein CP26; (**h**) abscisic acid receptor PYL2. Mean value ± standard deviation were represented, and different letters (a, b, c, d) indicate that they were significant differences at 0.05 level according to Duncan test.

To verify the accuracy of transcriptome results, eight genes were randomly selected from DEGs for qRT-PCR analysis. Genes were ethylene responsive transcription factor ERF054-like (Figure 6a), WRKY transcription factor 48 (Figure 6b), phenylalanine ammonia-lyase (Figure 6c), peroxidase 31 (Figure 6d), ABC transporter B family member 2 (Figure 6e), glutathione S-transferase U8 (Figure 6f), chlorophyll a-b binding protein CP26 (Figure 6g) and abscisic acid receptor PYL2 (Figure 6h). The results showed that the relative expression level of five genes in cucumber seedlings were significantly upregulated under cold stress, whereas the relative expression level of three genes were significantly downregulated. As expected, the expression of these genes verified by qRT-PCR showed the same expression patterns as in transcriptome results, thus confirming that the transcriptome data were reliable.

## 3. Discussion

### 3.1. Silicon Could Enhance the Cold-Tolerance of Cucumber Seedlings by Enhancing the Metabolism of Phenylpropanoid

Silicon plays a significant role in promoting crop growth and improving crop resistance to biotic and abiotic stresses [13,23]. Previous studies have shown significant differences in gene expression in cucumber leaves after silicon addition [24,25]. In this work, silicon induced the differential expression of 602 genes. These DEGs were mostly enriched in secondary metabolic processes dominated by phenylpropanoid metabolism. The phenylpropanoid metabolic pathway is a main metabolic pathway in crops [26], which produces a wide range of secondary metabolites such as phenols, lignans, flavonoids, and alkaloids under the action of various enzymes and plays a critical role in resistance to stress from adversity, phytochrome formation, and cellular lignification processes [17,27,28]. In our work, 16 genes involved in the metabolic process of phenylpropanoid were significantly expressed after the addition of silicon, indicating that silicon addition could enhance the secondary metabolic capacity of cucumber, which in turn enhanced its cold tolerance.

Three genes involved in bloom transport proteins (ABC transporters) were differentially expressed after silicon addition. In Arabidopsis, the ABC/LTP transporter proteins were found to be involved in bloom transport [29,30]. This indicates that the addition of silicon promotes bloom transport and enhances fruit bloom content, which is the same as the results of our previous study [6].

### 3.2. Rootstock Grafting Could Boost the Scavenging of ROS and the Synthesis of Phytohormones and Maintain the Stability of Membrane Structure to Enhance the Cold-Tolerance of Cucumber Seedlings

Grafting is an essential agronomic technique to enhance cold tolerance in cucumbers [2]. In this work, rootstock grafting induced differential expression of 596 genes (in ‘Huang Chenggen No. 2′-grafted cucumber) and 1349 genes (in ‘Yunnan figleaf gourd’-grafted cucumber). These DEGs were collectively enriched in membrane lipid metabolism and ROS metabolic pathways. Cold stress disrupts the balance of ROS metabolism, thereby allowing ROS to accumulate [31] and cause damage to the membrane structure. Alpha-linolenic acid helps to maintain membrane integrity under cold stress [32]. Glutathione metabolic processes scavenge ROS, maintain the balance of ROS in crops, and mitigate peroxidative damage to membrane structures [33]. We found that seven genes involved in α-linolenic acid metabolism were differentially expressed in both grafted cucumbers, whereas six genes (in ‘Huang Chenggen No. 2′-grafted cucumber) and eight genes (in ‘Yunnan figleaf gourd’-grafted cucumber) were involved in glutathione metabolism, suggesting that rootstock grafting can maintain the stability of membrane structure and boost the scavenging capacity of ROS in cucumber, thereby mitigating the damage caused by cold.

Compared with ‘Huang Chenggen No. 2′ rootstock, ‘Yunnan figleaf gourd’ rootstock induced DEGs that were more enriched in photosynthetic and hormonal signaling pathways. We found that fourteen genes involved in photosynthesis-related processes, seven genes involved in hormone signaling, and two genes involved in the MAPK signaling pathway were differentially expressed. We hypothesized that ‘Yunnan figleaf gourd’-grafted cucumber has a strong signal transduction ability and maintains stable photosynthesis to enhance the tolerance to the cold. A noteworthy finding is that in ‘Yunnan figleaf gourd’-grafted cucumber, the expression of four genes encoding bloom transporters (ABC transporters) increased, whereas the expression of only one gene was altered in ‘Huang Chenggen No. 2′-grafted cucumber. A previous study reported that grafting increased the upregulation of the bloom transporter protein gene CsABC in cucumber [24]. This suggests that rootstocks alter fruit bloom content by affecting the bloom transporter protein activity in cucumber scions, which we have proved before.

### 3.3. MYB, ERF and WRKY Play Transcription Factor Family Plays a Role in Cucumber Seedlings Response to Cold Stresses

With advances in technology, researchers have identified and analyzed many cold-responsive transcription factors, with WRKY, NAC, MYB, and AP2/ERF being the most abundant families of transcription factors induced under cold stress [19]. MYB transcription factors are closely related to the regulation of stress resistance in crops, e.g., overexpression of MYB44 significantly enhances the resistance of Arabidopsis to salt and drought stresses [34]. In this experiment, the expression of four MYB transcription factors, including MYB44, was upregulated under cold treatment, and the expression increased significantly after the addition of Silicon as compared with that in self-rooted cucumbers; these findings are consistent with the results of Zhu et al. [25]. It was speculated that these four transcription factors can enhance the cold tolerance of cucumber and their expression is influenced by silicon, and the addition of silicon increased their expression level, thus enhancing cold tolerance.

Ethylene is one of the endogenous hormones of plants, which responds to biotic and abiotic stresses in plants. ERFs belong to the AP2/EREBP (APETALA2/ethylene-responsive element binding proteins) transcription factor family and have been shown to be significant regulatory transcription factors in the ethylene-mediated stress response pathway [35,36]. In our work, ten ERFs were identified, indicating that ERFs are involved in the response of cucumber to cold stress. Over-expression of ERF054 significantly increased the tolerance of Arabidopsis to salt stress [37]. We found that the expression of ERF054 increased after cold treatment in both silicon-added and grafted cucumbers, and it is speculated that ERF054 can enhance the cold tolerance of cucumber and is regulated by both silicon and grafting.

The WRKY transcription factor family plays a role in plant response to abiotic stresses such as cold and drought [16,38]. In this research, WRKY48 was upregulated in grafted cucumbers from both rootstocks, suggesting that WRKY48 expression may be influenced by the rootstock and is positively correlated with cold tolerance in cucumber. WRKY70 plays a significant role in the resistance of Arabidopsis to abiotic stresses [39], whereas WRKY70 expression is also elevated in cucumber in response to salt stress [25]. WRKY70 was specifically expressed in ‘Yunnan figleaf gourd’-grafted cucumbers, whereas it disappeared in ‘Huang Chenggen No. 2′-grafted cucumbers. It is speculated that WRKY70 regulates the cold tolerance of grafted cucumbers and that its expression varies considerably between two types of rootstocks based on their ability to remove bloom.

## 4. Materials and Methods

### 4.1. Plant Materials and Cold Treatment

The experiment was conducted in the artificial climate chamber of Shandong Agricultural University from September 2020 to November 2020. The examined materials were ‘Xintaimici’ cucumber, the strong de-blooming rootstock ‘Huang Chenggen No. 2′ pumpkin, and the weak de-blooming rootstock ‘Yunnan figleaf gourd’ pumpkin. The cucumbers were used as both scions and self-rooted seedlings, and the two types of pumpkins were used as grafting rootstocks. Cucumber and pumpkin seeds were soaked in water for 4 and 8 h and germinated on a moist filter paper for 24 and 48 h at 28 °C, respectively, protected from light. Germinated pumpkin seeds were sown into trays filled with nursery substrate. For scions, cucumber seeds were sown 4 or 5 days later. When the first true leaves of the rootstock had unfolded, the cucumber seedlings were grafted using the ‘cuttage grafting’ method. The seedlings were cultivated in an internal artificial climate chamber at 28 °C/18 °C, 75–85% humidity, and 400 μmol·m^−2^·s^−1^ light intensity. When the first true leaves of self-rooted and grafted seedlings were fully expanded, six cucumbers were planted per pot in plastic pots (42 cm × 32 cm × 12 cm) filled with 6 L of Yamazaki Cucumber Formula Nutrient Solution, and the solution was renewed every 3 days. According to our previous studies, using Na_2_SiO_3_·9H_2_O as the silicon source and adding 0.5mM Na_2_SiO_3_·9H_2_O into the nutrient solution can effectively enhance the cold tolerance of cucumber. Thus, we set up the silicon addition (0.5 mM, Z+), non-silicon (Z) treatments for self-rooted cucumbers, the strong de-blooming rootstock ‘Huang Chenggen No. 2′-grafted cucumber (H), and the weak de-blooming rootstock ‘Yunnan figleaf gourd’-grafted cucumber (Y).

The seedlings were treated with cold (10 °C/5 °C, light and humidity conditions as mentioned above) when the third true leaf was fully unfolded. Each treatment was applied to 36 plants and repeated 3 times. Fresh leaves of cucumber seedlings were collected after 1 day of cold treatment for RNA extraction. Three biological replicates were established for each treatment, with six plants per replicate. All collected samples were immediately frozen in liquid nitrogen and stored at −80 °C until use.

### 4.2. Determination of Physiological Characteristics under Cold Stress

Fresh leaves of cucumber seedlings were collected after 7 days of cold treatment for determination of physiological characteristics. The malondialdehyde (MDA) content was measured using 2-thiobarbituric acid [40]. The method for determining chilling injury levels was slightly modified from that of Zhang et al. [41]. The chilling injury levels of different individual plants were investigated according to the following grading criteria: Level 5, the plant wilted and died; Level 4, the leaf margins of the heart leaves were dry, and the true leaves and cotyledons were completely dry; Level 3, the sum of the areas of dry spots on the cotyledons and true leaves was greater than 1/2 of the leaf area, and the leaf margins of the heart leaves were dry; Level 2, the sum of the areas of dry spots on the cotyledons and true leaves was about 1/2 of the leaf area, and the heart leaves were asymptomatic; Level 1, the dry spots on the cotyledons and true leaves were slightly dry, and the sum of the areas was less than 1/2 of the leaf area; At Level 1, the sum of dry spots on cotyledons and true leaves was less than 1/2 of the leaf area; at Level 0, the plants were growing normally without injury symptoms, and the chilling injury index was calculated according to the following formulas: chilling injury index = Σ (plants of different grade × grade)/[total plants × 5 (the maximum grade)]. For each determination of the physiological characteristics, three biological replicates were established for each treatment, with six plants per replicate.

### 4.3. Preparation and Sequencing of RNA Sequence Libraries

RNA sequence library construction and sequencing were conducted by Shanghai Majorbio Bio-pharm Technology Co., Ltd. (Shanghai, China). Total RNA from cucumber leaves was extracted from the tissue by using TRIzol^®^ reagent ((Invitrogen, Carlsbad, CA, USA) according to the manufacturer’s instructions (Invitrogen), and genomic DNA was digested by treatment with DNase I (Takara Bio, Tokyo, Japan). RNA quality was then determined by the Agilent 2100 Bioanalyzer and quantified using the ND-2000 spectrophotometer (NanoDrop Technologies, Beijing, China). Only high-quality RNA sample (OD260/280 = 1.8–2.2, OD260/230 ≥ 2.0, RIN (RNA integrity number) ≥ 6.5, 28S:18S ≥1.0, >2 μg) was used to construct the sequencing library.

The sequence transcriptome library was prepared following the instructions of the TruSeqTM RNA sample preparation kit from Illumina (San Diego, CA) by using 1 μg of total RNA. Briefly, mRNA was isolated according to the polyA selection method by using oligo(dT) beads and then fragmented by a fragmentation buffer. Next, double-stranded cDNA was synthesized using the SuperScriptTM double-stranded cDNA synthesis kit (Invitrogen, CA) with random hexamer primers (Illumina). The synthesized cDNA was then subjected to end repair, phosphorylation, and ‘A’ base addition according to Illumina’s library construction protocol. The libraries were size-selected for cDNA target fragments of 200–300 bp on 2% Low Range Ultra Agarose followed by PCR amplification using Phusion DNA polymerase (NEB) for 15 PCR cycles. After quantification with TBS380, the paired-end RNA sequence library was sequenced with the Illumina HiSeq xten/NovaSeq 6000 sequencer (2 × 150 bp read length).

### 4.4. Data Filtering and Mapping of Reads

The raw paired-end reads were trimmed and quality-controlled by SeqPrep (https://github.com/jstjohn/SeqPrep accessed on 29 December 2021) and Sickle (https://github.com/najoshi/sickle accessed on 29 December 2021) with default parameters. Clean reads were then separately aligned to the reference genome with the orientation mode using the TopHat (http://tophat.cbcb.umd.edu/ accessed on 29 December 2021, version 2.0.0) software. The mapping criteria of the bowtie was as follows: sequencing reads should be uniquely matched to the genome while allowing up to two mismatches, without insertions or deletions. The region of the gene was then expanded following depths of sites, and the operon was obtained. The whole genome was then split into multiple 15-kbp windows that shared 5 kbp. New transcribed regions were defined as more than two consecutive windows without an overlapped gene region, where at least two reads mapped per window were in the same orientation.

### 4.5. Screening of Differentially Expressed Genes (DEGs)

The differential expression of genes in cucumber leaves was analyzed using the DESeq2 (http://bioconductor.org/packages/stats/bioc/DESeq2/ accessed on 29 December 2021) software. First, high quality clean data were mapped to the cucumber genome database (https://www.ncbi.nlm.nih.gov/genome/?term=txid3659[Organism:noexp] accessed on 29 December 2021, version ASM407v2), and the number of reads in each sample was calculated. The expression levels of the annotated cucumber genes in all samples were then converted to fragments per kilobase per million mapped reads values. We used a false discovery rate (FDR) of 0.05 as the threshold for determining the significance of DEGs.

### 4.6. Functional Annotation and Enrichment Analysis of DEGs

The DEGs were mapped to the gene ontology (GO) database (http://www.geneontology.org/ accessed on 29 December 2021) to determine their contribution in biological processes, cellular components, and molecular functions. The DEGs were then mapped to the Kyoto Encyclopedia of Genes and Genomes (KEGG) pathway database (http://www.genome.jp/kegg/ accessed on 29 December 2021), and the genes were classified according to the pathway in which they are involved or the functions they performed. The *p*-value for significant enrichment in the KEGG pathways was calculated using the hypergeometric distribution test, and the *p*-value was then corrected by Benjamini and Hochberg’s multiple testing correction. The KEGG pathways were considered to be significantly enriched when the corrected *p*-value (FDR) was ≤ 0.05.

### 4.7. Transcription Factor Analysis and Screening

Transcription factor prediction and family analysis of genes were performed by analyzing the DNA-binding domain information of the genes using PlantTFDB 4.0 (http://planttfdb.cbi.pku.edu.cn/ accessed on 29 December 2021).

### 4.8. Quantitative Real-Time PCR (qRT-PCR) Analysis

Leave samples used for qRT-PCR were the same as those used for sequencing. Primer3Plus (http://primer3plus.com/cgi-bin/dev/primer3plus.cgi accessed on 29 December 2021) was used to design the primer sequences with the expected product size of 80–200 bp. The primers are listed in the Supplemental Table S5. Total RNA was extracted from the leaves by using Tranzol (TransGen Biotech, Beijing, China). The HiScript II One Step RT-PCR Kit (Vazyme Biotech, Beijing, China) was used to perform reverse transcription with 2μg of RNA according to the manufacturer’s instructions. The qRT-PCR was carried out using SYBR^®^ Green PCR Master Mix (Roche, CH, Switzerland) in a Rotor-Gene 3000 Real Time system (Qiagen, Hilden, Germany). qRT-PCR was performed on an ABI 75000 Real-Time PCR machine (Applied Biosystems, Foster City, CA, USA) with the following amplification program: 40 cycles of 94 °C for 15 s, 60 °C for 15 s, and 72 °C for 15 s. The melting curve was recorded after 40 cycles to verify the primer specificity. The relative quantization of gene expressions was calculated using the 2^−^^△^^△CT^ method [42]. Three biological replicates were established for each treatment, with six plants per replicate. 

### 4.9. Statistical Analysis

Microsoft Excel was used for data processing and visualization. Statistical analysis was performed using SPSS 26.0 (IBM, Chicago, IL, USA), and Duncan’s new complex range method was used to test for significant difference (*p* < 0.05).

## 5. Conclusions

The results of the physiological experiments demonstrated that both grafting and silicon addition enhanced the cold tolerance of cucumbers and that grafting enhanced cold tolerance more than silicon did. By analyzing the functions of DEGs and the involved metabolic pathways, we speculated that silicon could enhance the metabolism of phenylpropanoid in cucumber, whereas rootstock grafting could boost the scavenging of ROS and the synthesis of phytohormones and maintain the stability of membrane structure in cucumber. The difference in cold tolerance between the two rootstocks was attributed to the cold-tolerant rootstock used for grafting, which had stronger metabolic and signal transduction capacity and could maintain stable photosynthesis. We also identified cold responsive transcription factors such as *MYB*, *ERF*, and *WRKY* family members. Our results provide useful information for studying the response of cucumber to cold stress, and provide a starting point for further research.

## Figures and Tables

**Table 1:** Number of differentially expressed genes (DEGs) in cucumber leaves. Different treatments represent: self-rooted cucumber (Z), self-rooted cucumber with silicon addition (Z+), ‘Huangchenggen No. 2′-grafted cucumber (H) and ‘Yunnan figleaf gourd’-grafted cucumber (Y). The former is the treatment, whereas the latter is the control.

Compare	Total DEGs	Up	Down
Z+_vs_Z	433	274	159
H_vs_Z	412	264	148
Y_vs_Z	1217	679	538
Y_vs_H	305	107	198

## Data Availability

The data presented in this study are available in this article and the supplementary materials.

## Supplementary Materials

The following supporting information can be downloaded at: https://www.mdpi.com/article/10.3390/plants11030445/s1, Table S1: Sequencing quality statistics of cucumber leaf transcriptome under cold stress; Table S2: Statistical table of silicon-induced transcription factors; Table S3: Statistical table of transcription factors induced by grafting on ‘Huang Chenggen No. 2′; Table S4: Statistical table of transcription factors induced by grafting on ‘Yunnan figleaf gourd’; Table S5: Primer sequence of real-time fluorescent quantitative PCR.
