# Supplementary material for: Transcriptome and Physiological Analysis of Rootstock Types and Silicon Affecting Cold Tolerance of Cucumber Seedlings
Source: Plants (Basel). 2022 Feb 6;11(3):445. doi: 10.3390/plants11030445 (PMC8838756; doi:10.3390/plants11030445)
Supplement: Supplementary file 1 [file plants-11-00445-s001.zip › plants-1557273-supplementary.pdf]

plants

MDPI

Supplementary Materials

Table S1. Sequencing quality statistics of cucumber leaf transcriptome under cold stress.

Sample	Raw Reads	Clean Rreads	Q20%	Q30%	GC%	Total Mapped(%)
Z1	46241710	45569710	97.57	92.94	43.03	96.05%
Z2	43041650	42464898	97.57	92.89	43.54	96.26%
Z3	47441526	46897024	97.74	93.32	43.6	96.15%
Z+1	43726582	43257350	97.77	93.38	43.99	96.18%
Z+2	46267878	45690616	97.64	93.07	44.28	96.13%
Z+3	48080806	47532624	97.76	93.37	44.22	96.29%
H1	49053480	48529996	97.75	93.33	44.99	96.11%
H2	44898826	44429784	97.61	93.02	44.91	95.77%
H3	42480046	42000092	97.84	93.55	44.61	95.79%
Y1	47396360	46855292	97.52	92.94	44.29	95.84%
Y2	48157904	47623126	97.67	93.32	44.54	95.99%
Y3	51236556	50605962	97.61	93.2	44.58	95.75%

Table S2. Statistical table of Silicon induced transcription factors.

Family	Gene Name	Gene Description	Control FPKM	Treat FPKM	Expression
bHLH	LOC101219379	transcription factor bHLH67	6.45	11.38	up
	LOC101213736	transcription factor BEE 3-like	1.58	5.82	up
	LOC101212782	transcription factor bHLH94-like	1.13	2.49	up
	LOC101205864	transcription factor bHLH30-like	0.84	3.18	up
	LOC101204984	transcription factor bHLH62, transcript variant X2	1.48	0.85	down
MYB	LOC101212679	myb-related protein P-like	0.09	0.59	up
	LOC101219416	transcription factor WER-like	1.76	8.59	up
	LOC101203523	transcription factor MYB44-like	60.72	127.76	up
	LOC101214125	myb-related protein 308	0.11	1.06	up
ERF	LOC101206608	ethylene-responsive transcription factor ERF054-like	0.03	0.36	up
	LOC105435700	ethylene-responsive transcription factor ESR2-like	0	0.79	up
	LOC101219993	ethylene-responsive transcription factor 3	16.7	6.79	down
NAC	LOC105436223	NAC domain-containing protein 19	8.22	4.69	down
	LOC101206181	protein FEZ-like	16.91	7.88	down
	LOC101210422	transcription factor JUNGBRUNNEN 1	2.32	1	down
bZIP	LOC101223216	transcription factor TGA6, transcript variant X1	0.3	0.13	down
	LOC101214116	ocs element-binding factor 1	5.81	2.83	down
AP2	LOC101205762	AP2-like ethylene-responsive transcription factor AIL1	0.24	0.85	up
FAR1	LOC101220527	protein FAR1-RELATED SEQUENCE 2, transcript variant X1	0.43	1.13	up
HSF	LOC105435962	heat stress transcription factor B-2a-like	8.51	17.96	up
Others	LOC101216040	zinc-finger homeodomain protein 5	0.72	1.88	up
	LOC101204350	zinc-finger homeodomain protein 9-like	1.17	3.31	up
	LOC101212689	zinc finger protein CONSTANS-LIKE 6-like	3.31	12.98	up
	LOC101220917	dof zinc finger protein DOF3.6-like	1.64	3.7	up

	LOC101214816	zinc finger protein CONSTANS-LIKE 5-like, transcript variant X2	66.53	36.48	down
	LOC105435572	cyclic dof factor 1	6.47	3.23	down

Table S3. Statistical table of transcription factors induced by grafting on 'Huang Chenggen No. 2'.

Family	Gene name	Gene Description	Control FPKM	Treat FPKM	Expression
NAC	LOC101217253	NAC transcription factor 29-like	7.78	11.55	up
	LOC101206465	NAC domain-containing protein 100-like	2.58	4.92	up
	LOC101207407	NAC domain-containing protein 90-like	2.08	0.48	down
	LOC101212552	NAC domain-containing protein 67-like	31.44	15.61	down
	LOC105436223	NAC domain-containing protein 19	8.22	1.51	down
	LOC101206181	protein FEZ-like	16.91	7.19	down
	LOC101210422	transcription factor JUNGBRUNNEN 1	2.32	0.78	down
MYB	LOC101210268	transcription factor DIVARICATA	4.87	9.21	up
	LOC101214125	myb-related protein 308	0.11	0.85	up
	LOC101204148	myb-related protein Myb4-like	7.62	4.74	down
	LOC101209599	myb-related protein 305-like	9.33	4.12	down
	LOC101205761	vacuolar cation/proton exchanger 5, transcript variant X2	0.88	0.24	down
	LOC101212679	myb-related protein P-like	0.09	0.38	down
	LOC101214834	transcription factor MYB114-like, transcript variant X1	0.61	0.14	down
	LOC101204148	myb-related protein Myb4-like	7.62	4.74	down
	LOC101214240	transcription factor LAF1-like	1.97	0.6	down
ERF	LOC101206608	ethylene-responsive transcription factor ERF054-like	0.03	1.5	up
	LOC101211577	ethylene-responsive transcription factor ERF110-like	4.21	15.58	up
	LOC101213348	ethylene-responsive transcription factor ABR1-like	5.71	14.39	up
	LOC101209150	ethylene-responsive transcription factor ERF011-like	1.25	4.24	up
	LOC101213288	ethylene-responsive transcription factor ERF017	5.9	1.76	down
WRKY	LOC101205991	probable WRKY transcription factor 48	8.24	19.96	up
HSF	LOC101209621	heat stress transcription factor A-4d	7.74	3.27	down
bHLH	LOC101205943	transcription factor bHLH137	1.22	2.79	up
Others	LOC101216040	zinc-finger homeodomain protein 5	0.72	1.53	up
	LOC101212689	zinc finger protein CONSTANS-LIKE 6-like	3.31	11.85	up
	LOC101219003	zinc finger protein CONSTANS-LIKE 6	11.47	22.2	up
	LOC101204440	dof zinc finger protein DOF1.7-like	6.39	25.48	up
	LOC101206016	B-box zinc finger protein 18, transcript variant X1	35.37	17.14	down

Table S4. Statistical table of transcription factors induced by grafting on 'Yunnan figleaf gourd'.

Family	Gene Name	Gene Description	Control FPKM	Treat FPKM	Expression
MYB	LOC101216901	protein ODORANT1-like	1.83	3.4	up
	LOC101206909	myb-related protein 315-like	0.42	0.86	up

	LOC101206700	myb-related protein 308-like	1.51	3	up
	LOC101212775	transcription repressor MYB6-like	0	0.22	up
	LOC101216185	myb-related protein 330-like	3.92	5.6	up
	LOC101214125	myb-related protein 308	0.11	0.85	up
	LOC101212679	myb-related protein P-like	0.09	0.38	up
	LOC101213371	myb-like protein I	0	0.25	up
	LOC101216920	transcription factor MYB59-like	9.72	3.73	down
	LOC101204148	myb-related protein Myb4-like	7.62	4.74	down
	LOC101214240	transcription factor LAF1-like	1.97	0.6	down
NAC	LOC101209131	protein CUP-SHAPED COTYLEDON 3, transcript variant X2	1.07	3.31	up
	LOC101207407	NAC domain-containing protein 90-like	2.08	0.48	down
	LOC101212552	NAC domain-containing protein 67-like	31.44	15.61	down
	LOC105436223	NAC domain-containing protein 19	8.22	1.51	down
	LOC101211941	NAC domain-containing protein 21/22-like, transcript variant X2	31.31	18.23	down
	LOC101217627	NAC transcription factor 25	145.08	102.7	down
	LOC101214387	NAC domain-containing protein 100-like	1.39	0.97	down
	LOC101210422	transcription factor JUNGBRUNNEN 1	2.32	0.78	down
bHLH	LOC101203971	transcription factor bHLH74-like	0.13	0.46	up
	LOC101213736	transcription factor BEE 3-like	1.58	3.3	up
	LOC101205943	transcription factor bHLH137	1.22	2.79	up
	LOC101215588	transcription factor bHLH79	6.65	8.41	up
	LOC101212791	putative transcription factor bHLH041	0.42	1.01	up
	LOC101203107	transcription factor bHLH35-like	6.99	4.43	down
	LOC101205392	transcription factor bHLH128	43.71	23.38	down
WRKY	LOC101214847	probable WRKY transcription factor 21, transcript variant X2	0.14	0.54	up
	WRKY22	probable WRKY transcription factor 70-like	37.29	44.25	up
	WRKY52	probable WRKY transcription factor 68-like	0.87	1.55	up
	LOC101218177	probable WRKY transcription factor 48	1.42	2.73	up
	LOC101217296	WRKY transcription factor 55	1.83	1.09	down
ERF	LOC101206608	ethylene-responsive transcription factor ERF054-like	0.03	1.5	up
	LOC101213348	ethylene-responsive transcription factor ABR1-like	5.71	14.39	up
	LOC101206124	ethylene-responsive transcription factor ERF115	1.03	2.16	up
	LOC101207613	ethylene-responsive transcription factor CRF4	8.29	3.23	down
	LOC101219745	ethylene-responsive transcription factor TINY-like	6.78	3.57	down
HSF	LOC101208010	heat stress transcription factor A-6b-like, transcript variant X1	3.86	1.41	down
	LOC101209621	heat stress transcription factor A-4d	7.74	3.27	down
	LOC105435962	heat stress transcription factor B-2a-like	8.51	5.88	down
bZIP	LOC101203660	ocs element-binding factor 1	26.13	18.19	down
ARF	LOC101216825	auxin response factor 5, transcript variant X1	0.14	0.64	up
Others	LOC101216040	zinc-finger homeodomain protein 5	0.72	1.53	up
	LOC101216401	zinc finger protein CONSTANS-LIKE 9-like, transcript variant X1	0.92	2.64	up
	LOC101219003	zinc finger protein CONSTANS-LIKE 6	11.47	22.2	up
	LOC101204440	dof zinc finger protein DOF1.7-like	6.39	25.48	up
	LOC101215098	cyclic dof factor 3-like	0.84	1.18	up
	LOC101206938	dof zinc finger protein DOF5.7	1.78	1.08	down

LOC101206016	B-box zinc finger protein 18, transcript variant X1	35.37	17.14	down
LOC101208899	B-box zinc finger protein 18-like	7.64	4.39	down

Table S5. Primer sequence of real-time fluorescent quantitative PCR.

Gene_Name	Gene_Description	Primer Sequences (Forward/Reverse Primer)
LOC101206608	ethylene-responsive transcription factor ERF054-like	F:AATCACCACCCAATTCACCCATTCC<br>R:AGAGGCAGGAGAAGAAACAGAGGAG
LOC101218177	WRKY transcription factor 48	F:GCAGGGTGTGGTGTGAAGAAGAG<br>R:GAGGCTGTTGTTGGTGGATTCTGG
LOC101218178	phenylalanine ammonia-lyase	F:CGAGAGCAGCCATGTTGGTGAG<br>R:CCACGAAGAGGCAAGCAAGGAG
LOC101218179	peroxidase 31	F:GACATTCTCGCCCTTGCCACTC<br>R:TTTCGGTAAAGAGCCAGGAATCGTC
LOC101218180	ABC transporter B family member 2	F:TGGACGGATTGGAGTTGATGGAATG<br>R:TGACGCCGACAATGCCATACATC
LOC101218181	glutathione S-transferase U8	F:GGTTTCCGCTCAGGTTCGTATAGTC<br>R:CGGCACTTTCTGGTAAATTGGGTTG
LOC101218182	chlorophyll a-b binding protein CP26	F:GACTCAGCAAGAAGCCCGAAGAC<br>R:CCTCAGGGATGATGAAACCAGCAG
LOC101218183	abscisic acid receptor PYL2	F:CTTCAGGGTGGTGGGAGGAGAG<br>R:CGGTGTTCCCTTCTGGTATGTCAAC
	actin	F:CACGGGTATTGTTCTT<br>R:TCTTCTTGATGTCCCT
